# Translation and cross-cultural adaptation of heat strain score index (HSSI) into the Malay language

**DOI:** 10.1371/journal.pone.0281217

**Published:** 2023-02-22

**Authors:** Mei Ching Lim, Khamisah Awang Lukman, Nelbon Giloi, Mohammad Saffree Jeffree, Saihpudin Sahipudin Saupin, Zulkhairul Naim Sidek, Mohd Nazri Mohd Daud, Glynn Dexter Andreas

**Affiliations:** 1 Department of Public Health Medicine, Faculty of Medicine and Health Sciences, Universiti Malaysia Sabah, Kota Kinabalu, Sabah, Malaysia; 2 Centre for Occupational Safety & Health, Universiti Malaysia Sabah, Kota Kinabalu, Sabah, Malaysia; 3 Family Medicine Unit, Faculty of Medicine and Health Sciences, Universiti Malaysia Sabah, Kota Kinabalu, Sabah, Malaysia; 4 Department of Environmental and Occupational Health, Faculty of Medicine and Health Sciences, Universiti Putra Malaysia, Seri Kembangan, Selangor Darul Ehsan, Malaysia; Universiti Kebangsaan Malaysia, Faculty of Medicine, MALAYSIA

## Abstract

Concerns about the health and safety of working populations as well as preventive actions to reduce heat-related illnesses and fatalities have intensified as global warming and heatwaves continue to rise as a result of climate change. This study aimed to translate and culturally-adapted the translated Malay version of the Heat Strain Score Index (HSSI) questionnaire so that it can be utilized as a screening tool for heat stress among the Malay-speaking outdoor workers. The original English version of HSSI underwent forward-backward translation and was cross-culturally adapted into the Malay language by bilingual translators based on established guidelines. The content validation was reviewed by a six-member expert committee including the representative of outdoor workers. Face validation was carried out among 10 outdoor workers involved with various work tasks. Psychometric analysis was conducted based on a cross-sectional study among 188 workers who were eligible. Exploratory Factor Analysis (EFA) was used for construct validity while internal consistency reliability was performed using Cronbach’s alpha. The interclass correlation coefficient (ICC) was used to calculate the test-retest reliability. Both content and face validity were acceptable with the overall content validity index being 1.00, while the universal face validity index was 0.83. The factor analysis using varimax rotation extracted four factors which explained 56.32% of the cumulative percentage of variance and factor loading ranging from 0.415 to 0.804. The internal consistency reliability was acceptable with Cronbach’s alpha ranging from 0.705 to 0.758 for all the factors. The overall ICC value was 0.792 (95% CI; 0.764–0.801) which signifies good reliability. The findings from this study indicate that the Malay version of HSSI is a reliable and culturally-adapted instrument. Further validation is needed so that it can be used extensively assess the heat stress among susceptible Malay-speaking outdoor workers in Malaysia who are exposed to hot humid environments.

## Introduction

As global warming and heat waves continue to rise due to climate change, concerns regarding the health and safety of working populations, as well as preventive measures to minimize heat-related illnesses and fatalities, have increased. Outdoor workers are most susceptible to heat-related illnesses due to the challenging nature of their work, performed mainly outdoors under poor working conditions at temperatures over 35°C [1]. Heat stress not only increases the risk of heat-related illnesses but also intensifies the risk of accidental occupational injuries as a result of dizziness, exhaustion, lack of concentration, impaired decision-making or judgment, muscle weaknesses as well as clammy hands, and foggy safety goggles from the hot, humid working environment [2–4].

According to the National Institute of Occupational Safety and Health (NIOSH), heat stress is defined as the accumulation of heat the body experiences, which is measured as the total of the heat generated in the body (metabolic heat) in addition to environmental heat gained from the surroundings including work settings, but deducting the heat lost from the body to the environment [5]. A considerable radiant heat load can further aggravate heat stress from direct sunbeams, internal body heat, and an increased metabolic rate, all of which are caused by physical and intense activity [4].

Most workers and employers in Malaysia still lack awareness of the potential hazard of working in hot weather, especially as the ambient temperature continues to rise due to climate change [6]. According to an online survey conducted by Zander & Mathew (2019) among Malaysian adults, almost 99% of the 514 respondents claimed to experience heat stress symptoms at some point, which resulted in a 50% reduction in work capacity and a 29-days per year reduction in productivity [7]. Hence, employers must understand and prepare mitigating measures to face climate change that may affect their employees’ health, safety, and welfare in accordance with the Occupational Safety and Health Act (OSHA) 1994 [8].

A heat stress index incorporates a few elements, namely personal, physiological, and thermal environmental parameters, into a single value to measure workers’ heat stress exposure [9, 10]. Many heat indices have been recommended for various hot and humid environments, but no single heat index has gained universal recognition based on the various parameters involved [11, 12]. Heat indices, which are low-cost, non-invasive, simple to use, as well as based on an individual’s subjective experience, observation, and self-reported symptoms, have also been developed to assess heat stress responses [13, 14]. These indicators are thought to be essential in overcoming the limitations of invasive and precise field measurements.

One of them is the heat strain score index (HSSI), a questionnaire developed to determine the risk factors for heat strain [15, 16]. The individual’s observations, perceptions, and judgments about heat-related symptoms and behaviours are used to calculate this index [15, 16]. HSSI has been frequently applied over the years to analyze and compare heat strain responses in a variety of occupations, including bakers [17], municipal workers [18], open-pit miners [19], and steel workers [20].

Despite its widespread use, there is no translation performed on the HSSI questionnaire into the Malay language that has been published to the author’s knowledge. The translated Malay version HSSI questionnaire is important to assess the workers’ perception and observation of their work environment as well as their perceived symptoms at work which will also assist in the control and preventive measures at work. The main objective of this research is to translate, adapt and validate the translated Malay version of the HSSI questionnaire so that it can be utilized as a screening tool for heat stress and heat strain among the Malay-speaking high-risk working population in Malaysia before a further invasive assessment is conducted.

## Materials and methods

### HSSI questionnaire

Validated Heat Strain Score Index (HSSI) to assess responses based on respondents’ perceptions of their workplaces was originally developed by Dehghan et al. (2015) [15]. The HSSI scale comprises 18 items. These include 12-item observational questions and 6-item perceptual questions. These questions are related to workplace ambient (air temperature, humidity sensation, temperature of adjacent surfaces, air flow), perceived symptoms at work (intensity of thirst, sweating, and suffering from the heat, level of fatigue, heat stress symptoms), workplace settings (dimensions of the working space, types of working environment, ventilation system), working attire (types of clothes, colour, and material of working attire, personal protective equipment), the intensity of physical activity and body postures at work. Primary scores are allocated for each answer to every question, unlike the standardized four- or five-point Likert scale, commonly used [21]. The primary score of each question is multiplied by the effect coefficient to obtain the final score. The final scores will be added to become the total score and are then divided into three heat strain risk categories: (i) no or low heat strain (total score less than 13.5); (ii) likelihood of heat strain (total score between 13.6 to 18); and (iii) definitive heat strain (total score more than 18).

All the items in the original HSSI questionnaire have satisfactory factor loading of more than 0.40 (ranging from 0.44 to 0.90) and good internal consistency reliability with Cronbach’s alpha of more than 0.70 (ranging from 0.78 to 0.85) from its development process [15]. Even though the previous study in Malaysia utilized the HSSI questionnaire [18], no published Malay version of HSSI was recorded; hence, it would be more appropriate for the HSSI questionnaire to be translated into Malay, as most Malaysians can comprehend and communicate in the Malay language despite their different cultural backgrounds. The Malay version of the questionnaire would assist the respondents in sharing their actual experiences, judgments, and thoughts.

This study comprised two phases: phase 1 involves translating and adapting the original English version of HSSI into the Malay language based on the Guideline for Establishing Cultural Equivalency of Instruments [22] and the outline of the Questionnaire Development and Translation Process [23]. The researchers must understand the fundamental ideas, factors, and methodological issues involved, particularly in the quality of translation and the equivalence of results in different cultural and ethnic groups, because cross-cultural research has exceptional intricacies [24]. Phase 2 included Validation of the translated Malay version of HSSI, namely content validation, face validation, and psychometric analysis.

### Translation and adaptations

#### Forward translation

The initial translation from English to Malay was conducted by two independent translators who are well versed in both Malay and English and have background knowledge regarding Occupational and Environmental Health. This is to ensure the item and meaning of the translation resemble the original questionnaire. Each of the translators produced separate reports. The translated questionnaires from both translators were then compiled into a single translated questionnaire. Differences in word choice between the two translators were discussed until consensus was reached on which word to use.

#### Backward translation

The finalized translated Malay questionnaire was then independently translated back to English by another two different translators with bilingual proficiency and background knowledge of Occupational and Environmental Health. Both these translators were not exposed to the original English Version of HSSI. This step is important to ensure the accuracy of the translation when it undergoes backward translation, as well as to identify any unclear or unsuitable wordings in the initial translation. Discrepancies between forward and backward translation were discussed between the researcher and all the translators. This is vital to ensure there were no differences in the wordings between the original items and the backward-translated version and assuring the translated questionnaire is appropriate for the Malaysian population, particularly the working community.

### Validation of the translated Malay questionnaire

#### Content validation by expert committee

Content validity refers to the extent to which a collection of items is considered together to establish an acceptable operational definition of a construct, mainly involving judgment or evaluation through careful conceptualization and domain analysis, especially in cross-cultural research [23, 24]. Methodological challenges include adapting the translated questionnaires into an understandable and culturally acceptable form among the local community while preserving the meaning of the original questionnaire [24].

Each committee member was given a thorough briefing on the content validation process, which was conducted using the widely used content validity index (CVI) [25]. The content validation form was emailed to all the experts, and they were given two weeks to complete the evaluation. Even though it would be more appropriate to conduct the content validation face-to-face approach, a non-face-to-face approach was chosen because of the COVID-19 pandemic, as well as to balance the cost and time challenges of gathering all the experts together [26]. The experts scored each item for relevance on a 4-point scale (1 = not relevant, 2 = somewhat relevant, 3 = quite relevant, and 4 = highly relevant).

#### Face validation (pilot-testing finalize draft of translated questionnaire)

Face validity is defined as the degree to which the raters evaluate the items of an instrument to be appropriate for the intended construct and objectives [26]. A face validation form comprising rating scores for clarity and comprehensiveness was developed to score individual items of the translated Malay version of HSSI. This ensures that the intended reviewers understand the task and evaluate accordingly. The ’clarity’ of commands, languages, and phrases applies to whether there were any inconsistencies or various ways to interpret the items, whereas the ’comprehensibility’ of commands, languages, and phrases indicates whether the words or sentences of the constructed items may well be easily understood by the reviewers [26]. It is of utmost importance to establish face or response process validity to support the overall validity of the translated Malay HSSI.

A small group of 10 reviewers was selected to rate and comment on the complete set of questionnaires. For face validation, a minimum of ten raters is required [26–29]. The reviewers included various groups of workers working in a hot environment or under the hot sun, including construction workers (two), landscape workers (three), outdoor cleaners (three), and rubbish collectors (two). Interviews based directly on the questionnaire were conducted among three illiterate workers. After reviewing and rating the questionnaire, the respondents were interviewed on their thoughts, understanding, and feedback on difficulties in answering the questionnaire, especially the suitability of the choice of words according to the community’s cultural context. This is to ensure the sampled respondents have a clear understanding, with no confusion, and that the translated HSSI items contained the same meaning or concept as the original questionnaire. These steps were considered crucial to confirm comprehensibility and to fulfill the face validity or response process validity before the pilot study.

#### Field study or psychometric analysis

After the finalized draft questionnaire was ready, a cross-sectional field study was conducted from January 2022 to March 2022 among the working population, which shared similar characteristics and activities to the intended sample population. The respondents were different from the respondents from the pilot testing. The outdoor workers were selected through purposive sampling from various occupations exposed to sunlight or the hot environment during their working hours. They include construction workers, landscape workers, sweepers, cleaners, and rubbish collectors. The researcher approached them and invited them to participate in the field study. Workers who consented were then screened for eligibility based on the inclusion and exclusion criteria of the study. Workers who are more than 18 years old and exposed to heat or sun during their eight hours shift per day were included while workers who were pregnant and with underlying co-morbid like heart disease, autoimmune disorders, thyroid disease or mental illnesses were excluded. Self-administered questionnaires were given to the participants, and interviews were conducted strictly based on the questionnaire for illiterate workers. One hundred and eighty-eight outdoor workers were recruited for the field study. A minimum sample of 180 respondents was needed with a subject-to-item ratio of 10:1, as recommended by Hair et al. (2014) [30]. Returned questionnaires were examined for missing data or duplicated answers. Clarifications were made to questions that had more than two answers to ensure respondents chose the most appropriate answers to their understanding.

*Construct validity*. Construct validity refers to the extent to which the translated Malay version of HSSI was correlated to other measures based on hypothetically derived, pre-defined hypotheses [31]. The construct validity was conducted by performing the exploratory factor analysis (EFA).

*Internal consistency*. Internal consistency or reliability refers to how closely all test items assess the same notion or construct and is thus related to how closely the test items are related to one another. Before a test is used for research or examination purposes, internal consistency should be established to assure validity. Internal consistency defines the extent to which all the test items assess the same notion or construct and is thus related to how closely the test items are related to one another [32].

*The intraclass correlation coefficient (ICC)*. ICC refers to the correlation within a class of variables or items for repeated measurements, and it was conducted to assess the test-retest reliability of the items [33]. Test-retest reliability is usually used to evaluate the degree of consistency to meet the objective of validating a questionnaire design, particularly during the initial pilot or field testing [34]. Data were collected from the participants using the same questionnaire three to four weeks after the first questionnaires were distributed.

### Statistical analysis

Microsoft Excel was used for initial data collection and cleaned before data analysis was performed using IBM SPSS statistical package version 28.0. For descriptive statistics, normally distributed continuous variables were presented as mean and standard deviation, while categorical variables were presented as frequency and percentage. Both content validity index (CVI) and Face Validity Index (FVI) were computed using Microsoft Excel.

#### Content validity

The content validity index (CVI) score was computed for both the item level (I-CVI) and the scale level (S-CVI) [35]. Before the calculation of I-CVI, the score of each item was re-coded from the 4-point relevance scale into two new categories in which items scored either 1 or 2 were allocated into the "not relevant" category with ’0’ point each while items scored either 3 or 4 were allocated into the "relevant" category with ’1’ point each. The accumulated points were then divided by the number of experts (six) to obtain the I-CVI value for each item.

The S-CVI is the average of the I-CVIs for all of the scale items. There are two approaches for calculating S-CVI: (i) calculation of the average of the I-CVI scores for all items on the scale (S-CVI/Ave); and (ii) the proportion of items on the scale that achieve universal agreement (a relevance scale of 3 or 4 by all experts or score ’1’ after re-coding) (S-CVI/UA) [35]. The definition and formula of the CVI indices are summarized in Table 1.

**Table 1:** Definition and formula for content validation indices and face validation indices.

Indices	Definition	Formula
**Content Validation Indices**		
I-CVI	The number of items given a relevance rating of 3 or 4 divided by the number of experts	I-CVI = (agreed item) / (number of expert)
(Item-level Content Validity Index		
S-CVI/Ave	The average of the I-CVI scores for all items on the scale or the average of proportion relevance judged by the experts. The proportion relevant is the average of relevance rating by individual expert.	S-CVI/Ave = (sum of I-CVI scores) / (number of item)
(Scale-level Content Validity Index based on the average method)		S-CVI/Ave = (sum of proportion relevance rating) / (number of expert)
S-CVI / UA	The proportion of items on the scale that achieve a relevance scale of 4 or 4 by all experts. Universal agreement (UA) is given as ‘1’ when the item achieved 100% agreement among the experts. If not, the UA score is given as ‘0’.	S-CVI/UA = (sum of UA scores) / (number of item)
(Scale-level Content Validity Index based on the universal agreement method		
Source: (Davis, 1992; Lynn, 1986; Polit & Beck, 2006; Yusoff, 2019)		
**Face Validation Indices**		
I-FVI	The number of items given a clarity and comprehensibility rating of 3 or 4 divided by the number of reviewer	I-FVI = (agreed item) / (number of reviewer)
(Item-level Face Validity Index		
S-FVI/Ave	The average of the I-CVI scores for all items on the scale or the average of proportion clarity and comprehensibility judged by the raters. The proportion clarity and comprehension are the average of rating by individual rater.	S-FVI/Ave = (sum of I-CVI scores) / (number of item)
(Scale-level Face Validity Index based on the average method)		
		S-FVI/Ave = (sum of proportion relevance rating) / (number of reviewer)
S-FVI / UA	The proportion of items on the scale that achieve a clarity and comprehension scale of 3 or 4 by all reviewers. Universal agreement (UA) is given as ‘1’ when the item achieved 100% agreement among the reviewers. If not, the UA score is given as ‘0’.	S-FVI/UA = (sum of UA scores) / (number of item)
(Scale-level Face Validity Index based on the universal agreement method		

*Source: (Ozair et al., 2017, Yusoff, 2019)

#### Face validity

Face validity can be represented by the face validity index (FVI), which is executed and calculated based on the measures outlined by previous studies [26–29]. The degree of clarity and comprehensibility was rated according to the 4-point scale (1 –not clear and not understandable, 2 = somewhat clear and understandable, 3 = clear and understandable, and 4 = very clear and understandable).

Before the calculation of the face validity index (FVI), the score of each item was re-coded from the 4-point clarity and comprehensibility scale into 2 new categories in which items scored either 1 or 2 were allocated into the "not clear and understandable" category with ’0’ point each while items scored either 3 or 4 were allocated into the "clear and understandable" category with ’1’ point each. The FVI score was computed for both the item level (I-FVI) and the scale level (S-FVI) [26]. The accumulated points were then divided by the number of raters (10) to obtain the I-FVI value for each item.

The S-FVI is the average of the I-FVI scores for all of the scale items. There are two approaches for calculating S-FVI: (i) calculation of the average of the I-FVI scores for all items on the scale (S-FVI/Ave); and (ii) the proportion of items on the scale that achieve universal agreement (a clarity and comprehensibility scale of 3 or 4 by all reviewers or score of ’1’ after re-coding) (S-FVI/UA) [26]. The definition and formula of the FVI indices are summarized in Table 1.

#### Construct validity

The construct validity was conducted by performing the exploratory factor analysis (EFA) using IBM SPSS Statistical Tool. The initial analysis included assessing the assumptions of factor analysis followed by generating and examining Bartlett’s test of sphericity, Kaiser-Meyer-Olkin (KMO) measure of sampling adequacy, and communalities [36]. In order to determine the components that underlie each of the 18 items, factor extraction was carried out using principal axis factoring after the appropriateness of the initial analysis. The following analysis was used to determine the number of components to be retained: (i) eigenvalue larger than one rule; (ii) examining the scree plot test; and (iii) the need for at least 50% of the cumulative percentage of variance [30, 37]. The factors obtained were then rotated using varimax, an orthogonal rotation that attempts to minimize the number of variables that have high loadings on a factor and, thus, improves the factors’ interpretability [36, 37]. At least three measured items are required to statistically identify a component with loadings greater than 0.3 [38, 39].

#### Internal consistency

Cronbach’s alpha was performed to approximate the internal consistency of each component or factor generated in PCA using IBM SPSS. The lowest accepted Cronbach’s alpha values were considered at 0.70, which verifies that the correlations among items on the same components are acceptable [32].

#### Interclass correlation coefficient (ICC)

ICC and their 95% confidence intervals were analysed in IBM SPSS Version 28.0, including mean-rating (k = 2), absolute agreement, and two-way mixed-effects model reliability [40]. Values between 0.75 and 0.9 and > 0.90 signify good and excellent reliability, respectively, based on the 95% confidence interval of the ICC estimate [40].

### Ethical considerations

The original HSSI questionnaire with the calculation is shared by the researcher/developer in PDF format and set in the public domain with open access [15]. Additional permission from the author/developer of the original HSSI questionnaire was also obtained. Ethical approval was obtained from the Ethical Committee of the Faculty of Medicine and Health Sciences, Universiti Malaysia Sabah (UMS) [Approval Code: JKEtika 3/21 (18)]. The field study was conducted in accordance with the Declaration of Helsinki and any additional protocol or requirement from the UMS Ethical Committee. Informed consent was obtained from every participant prior to the study, either written consent which was signed by the participants or verbal consent which was witnessed by the workers’ supervisors or leaders. All the necessary information was explained to the respondents thoroughly, including the nature and purpose of the study, the procedures, and the tool used. The participants were assured that the collected data and information were strictly for research purposes and would not be used for any other purpose or disclosed to others without the participant’s permission. No personal information was revealed in the reports and publications. Participation in the study was entirely voluntary, with no payment given.

## Results and discussion

### Translation and adaptation

#### Forward translation

The translated questionnaires from both translators were compiled into a single translated questionnaire. Differences in word choice between the two translators were discussed until consensus was reached on which suitable word to use. Amendments were made to ensure the words in the translated questionnaire were appropriate for the Malaysian population, particularly the outdoor working community.

#### Backward translation

The finalized translated Malay questionnaire was then independently translated back to English by another two different translators with bilingual proficiency and background knowledge of Occupational and Environmental Health. Discrepancies between forward and backward translation were discussed between the researcher and all the translators. The meaning of most of the items which underwent backward translation was found to correspond to the original English questionnaire except item 1, item 4, item 8, and item 9. All four translators were sought for suggestions, and the researchers then worked on rephrasing the identified items.

■ In item 1, the translators decided to use the word ’*panas*’ instead of ’*suam*,’ which was the direct translation to ’warm’ as it is more understandable to describe the ambient temperature in the local context. Hence, the phrases’ slightly warm’, ’warm,’ and ’very warm’ were translated to ’*sedikit panas*,’ ’*panas*,’ and ’*sangat panas*’ to differentiate the different ambient temperatures.
■ In item 4, the ‘…*pengaliran keluar masuk udara sejuk*….’ was rephrased to ‘… *peredaran udara sejuk*…’ for the phrase ‘…cold weather circulation… ‘.
■ For item 8, the word ’*intensiti*’ was changed to ’*tahap’* as ’*intensiti*’ is not commonly used in Malay, and the workers may have problems understanding the word.
■ For item 9, all the translators agreed to use the word ’*terkesan*’ instead of ’*geram*,’ which was deemed more appropriate among the local community than the original word ’annoyed.’

The preliminary version of the Malay version of HSSI was then finalized.

### Content validation

An expert committee comprising six members comprising of two Occupational Health Doctors, an Environmental Health Officer, a Public Health physician, a Family Medicine Specialist, and a representative of the outdoor workers were consulted to review both versions of the translations and quantitatively evaluate the questionnaires.

All of them rated all items as 1.00 for the translated questionnaire. Hence the calculated I-CVI for all of the items and the S-CVI were 1.00. The relevance ratings on the item scale by the six experts and the calculation of different CVI indices for the Malay version of the HSSI questionnaire are illustrated in Table 2. This meant the content validation of the Malay version of HSSI was acceptable [35] and no item was further edited or removed.

**Table 2:** The relevance ratings on the item scale by the six experts and the calculation of different CVI indices for the translated Malay version of HSSI.

*Heat Strain Score Index	Expert 1	Expert 2	Expert 3	Expert 4	Expert 5	Expert 6		Experts in agreement	Universal Agreement (UA)	Item-level Content Validity Index (I-CVI)
Q1	1	1	1	1	1	1		6	1	1.00
Q2	1	1	1	1	1	1		6	1	1.00
Q3	1	1	1	1	1	1		6	1	1.00
Q4	1	1	1	1	1	1		6	1	1.00
Q5	1	1	1	1	1	1		6	1	1.00
Q6	1	1	1	1	1	1		6	1	1.00
Q7	1	1	1	1	1	1		6	1	1.00
Q8	1	1	1	1	1	1		6	1	1.00
Q9	1	1	1	1	1	1		6	1	1.00
Q10	1	1	1	1	1	1		6	1	1.00
Q11	1	1	1	1	1	1		6	1	1.00
Q12	1	1	1	1	1	1		6	1	1.00
Q13	1	1	1	1	1	1		6	1	1.00
Q14	1	1	1	1	1	1		6	1	1.00
Q15	1	1	1	1	1	1		6	1	1.00
Q16	1	1	1	1	1	1		6	1	1.00
Q17	1	1	1	1	1	1		6	1	1.00
Q18	1	1	1	1	1	1		6	1	1.00
								**S-CVI /Ave (based on I-CVI)**		1.00
**Proportion relevance**	1	1	1	1	1	1		**S-CVI /UA**	1	
**Average proportion of items judged as relevance across 6 experts S-CVI/Ave (based on proportion relevance)**							1.00			

* Recoding of relevance scale from questionnaire

•0 (relevance scale of 1 or 2)

• 1 (relevance scale of 3

### Face validation

Face validity is defined as the degree to which the raters evaluate the items of an instrument to be appropriate for the intended construct and objectives [26]. Before tabulating the face validity, inter-rater reliability, which is to evaluate the consistency or agreement between the 10 reviewers/raters in their ratings were also conducted using intraclass correlation coefficient (ICC) with mean rating (k = 10), absolute-agreement, and two-way mixed effects model. The overall ICC value was 0.887 (95% CI; 0.789–0.951), which signifies good inter-rater reliability.

The ten reviewers’ clarity and comprehension ratings on the item scale and the calculation of different Face Validity Index (FVI) indices for the questionnaire, including translated Malay version of HSSI, are tabulated in Table 3. The calculated I-FVI for all of the items and the S-FVI were equal to or more than the minimum value of 0.83 [26, 28]. Hence, the face validation for the translated Malay version of HSSI is acceptable.

**Table 3:** The clarity and comprehensibility ratings on item scale by the 10 reviewers and the calculation of different FVI indices for the translated Malay version of HSSI.

*Heat Strain Score Index (HSSI)	R1	R2	R3	R4	R5	R6	R7	R8	R9	R10		Reviewers in agreement	Universal Agreement (UA)	Item-level Face Validity Index (I-FVI)
Q1	1	1	1	1	1	1	1	1	1	1		10	1	1.00
Q2	1	1	1	1	1	1	1	1	1	1		10	1	1.00
Q3	1	1	1	1	1	0	1	1	1	1		9	0	0.90
Q4	1	1	1	1	1	1	1	1	1	1		10	1	1.00
Q5	1	1	1	1	1	1	1	1	0	1		9	0	0.90
Q6	1	1	1	1	1	1	1	1	1	1		10	1	1.00
Q7	1	1	1	1	1	1	1	1	1	1		10	1	1.00
Q8	1	1	1	1	1	1	1	1	1	1		10	1	1.00
Q9	1	1	1	1	1	1	1	1	1	1		10	1	1.00
Q10	1	1	1	1	1	1	1	1	1	1		10	1	1.00
Q11	1	1	1	1	1	1	1	1	1	0		9	0	0.90
Q12	1	1	1	1	1	1	1	1	1	1		10	1	1.00
Q13	1	1	1	1	1	1	1	1	1	1		10	1	1.00
Q14	1	1	1	1	1	1	1	1	1	1		10	1	1.00
Q15	1	1	1	1	1	1	1	1	1	1		10	1	1.00
Q16	1	1	1	1	1	1	1	1	1	1		10	1	1.00
Q17	1	1	1	1	1	1	1	1	1	1		10	1	1.00
Q18	1	1	1	1	1	1	1	1	1	1		10	1	1.00
												**S-FVI /Ave (based on I-FVI)**		0.98
**Proportion clarity and comprehensibility**	1	1	1	1	1	0.94	1	1	0.94	0.94		**S-FVI /UA**	0.83	
**Average proportion of items judged as clear and understandable across 10 reviewers S-FVI/Ave (based on proportion clarity and comprehensibility)**											0.98			

* Recoding of clarity and comprehensibility scale from the questionnaire

• 0 (relevance scale of 1 or 2)

• 1 (relevance scale of 3 or 4

### Psychometric analysis

#### Sociodemographic and work characteristics of participants

A field study was conducted among the outdoor working population, which shared similar characteristics and activities to the intended sample population for the psychometric testing. Two hundred participants consented to be involved in the study, but 12 responses had to be excluded due to incompleteness. Hence, only 188 questionnaires were analyzed.

Most participants were male (65.4%) and were of Bajau ethnicity (22.3%). The age range of the respondents was from 17 to 62 (mean 35.7 ± 9.4) years old. 18.1% did not have formal education, while the remaining 81.9% had at least primary school level education. 75% of the participants earned a household income of less than RM2000 per month. Most participants were cleaners and rubbish collectors (23.9%), followed by sweepers (21.3%), landscape workers (18.1%), supervisors or guards (14.9%), and construction workers (14.4%). All the participants worked a minimum of eight hours a day, and 79.2% were exposed to sunlight or a hot environment for at least six hours per day. Most of the workers (44.1%) were usually walking, while 30.3% were standing with high mobility at work. The summary of the participants’ sociodemographic and work characteristics is tabulated in Table 4.

**Table 4:** Summary distribution of the sociodemographic and work characteristics of outdoor workers for field study (N = 188).

Factors	Frequency, n	Percentage (%)	Mean (SD)
**Sociodemographic**			
Gender			
Male	123	65.4	
Female	65	34.6	
Age Group			35.7 (± 9.4)
≤ 36	118	62.8	
>36	70	37.2	
Ethnicity			
Bajau	42	22.3	
Kadazan/Dusun	41	21.8	
Malay	38	20.2	
Indian	17	9.0	
Bugis	12	6.4	
Brunei	11	5.9	
Others	27	14.4	
Marital Status			
Single	57	30.3	
Married	126	67.0	
Divorced	3	1.6	
Widow/Widower	2	1.1	
Level of Education			
None	34	18.1	
Primary School	47	25.0	
Secondary School	82	43.6	
Diploma/Degree	25	13.3	
Household Income			
< RM1000	24	12.8	
RM1000-1999	117	62.2	
> RM2000	47	25.0	
**Work**			
Types of Job			
Cleaner and rubbish collector	45	23.9	
Sweeper	40	21.3	
Landscape workers	34	18.1	
Supervisors / Guards	28	14.9	
Construction workers	27	14.4	
Others	14	7.4	
Working environment			
Outdoor	162	86.2	
Outdoor and Indoor	26	13.8	
Exposure to sun or hot environment (hours/day)			
≤ 5	39	20.7	
6–8	147	78.2	
> 8	2	1.1	
Activity at work			
Usually walking	83	44.1	
Usually standing with high mobility	57	303	
Usually standing with low mobility	25	13.3	
Usually sitting	23	12.2	

#### Exploratory factor analysis for construct validity

The Kaiser-Meyer-Olkin measure for sampling adequacy was 0.812, which surpassed the recommended minimum value of 0.70 (Kaiser, 1974). Bartlett’s test of sphericity (test of the factorability of the correlation matrix) was also significant [χ^2^(153) = 1146.74, p<0.001], which indicates the items were correlated and the correlation matrix of the variables in the dataset diverges significantly from the identity matrix [30, 37]. The communality values, which assess how well each variable is explained by the factors are acceptable with values ranging from 0.415 to 0.804. Assumptions of multivariate normality and linearity were fulfilled. Multicollinearity is not present as Variance Inflation Factor (VIF) analysed using linear regression was < 10 (range:1.187 to 2.486). Thus, factor analysis was conducted using principal axis factoring with all 18 items. Factor loading was set at 0.30 to suppress all loadings less than 0.30.

Four factors were identified with eigenvalues of more than 1, which accounted for 56.32% of the cumulative percentage of variance. The first factor described 28.25% of the variance, the second factor explained 12.44% of the variance, while the third and fourth factors described 9.54% and 6.09% of the variance, respectively. After varimax rotation, all the components had at least three items with factor loadings greater than 0.40. Therefore, no item was removed. The factors extracted were labelled as:

- Factor 1: Physical activity and perceived symptoms at work
- Factor 2: Observation and Perception of workplace ambient
- Factor 3: Working attire and personal protective equipment
- Factor 4: Observation of workplace setting

Table 5 summarizes the results of the items, factor loadings, communalities, and eigenvalue for the rotated factors.

**Table 5:** Factor loadings and communalities based on the principal axis factoring with varimax rotation for 18 items and Cronbach’s alpha on the HSSI-Malay version questionnaire (N = 188).

Items	Factor Loading				Communality
	Factor 1	Factor 2	Factor 3	Factor 4	
Factor 1: Physical activity and perceived symptoms at work					
8. Intensity of thirst at work	0.825				0.804
7. Level of fatigue at work	0.644				0.552
18. Heat stress symptoms at work	0.595				0.487
9. Intensiveness of suffering from heat	0.559				0.509
5. Intensity of physical activity at work	0.546				0.461
6. Amount of sweating at work	0.541				0.626
17. Body posture at work	0.457				0.512
Factor 2: Observation and perception of workplace ambient					
1. Air temperature in the workplace		0.766			0.599
3. Temperature of adjacent surfaces		0.715			0.578
2. Humidity level in the workplace		0.681			0.572
4. Flow of air in the workplace		0.531			0.438
Factor 3: Working attire and personal protective equipment					
13. Kind of clothes at work			0.523		0.469
16. Personal protective equipment at work			0.487		0.420
14. Colour of working attire			0.483		0.419
15. Material of working attire			0.467		0.415
Factor 4: Observation of workplace setting					
10. Size of working space within the building				0.866	0.760
11. Ventilation system in the workplace				0.650	0.691
12. Types of working environment				0.635	0.548
Eigenvalue	5.085	2.239	1.718	1.096	
% of variances	28.25	12.44	9.54	6.09	
Cronbach’s alpha	0.758	0.753	0.737	0.705	

*Cronbach’s alpha for internal consistency*. Cronbach’s alpha was performed to approximate the internal consistency of the overall value and each factor. The overall Cronbach’s alpha was 0.776, while Cronbach’s alpha for each factor ranged from 0.705 to 0.758. Hence, the internal consistency reliability is acceptable and verifies that the correlations among items on the same factors were also acceptable [32].

*The intraclass correlation coefficient (ICC) for test-retest reliability*. The test-retest reliability was conducted on the total HSSI scoring using ICC with mean rating (k = 2), absolute-agreement, and two-way mixed effects model on 178 participants at 3–4 weeks intervals. Thirty-six participants were uncontactable and had to be dropped out from the retest. The remaining 152 participants were still a good sample size for test-retest reliability [40]. The overall ICC value was 0.792 (95% CI; 0.764–0.801), which signifies good reliability.

## Discussion

This study executed the step-by-step measures suggested in the literature to develop a reliable and culturally-adapted Malay version of HSSI suitable for the working population in Malaysia. The Malay version of HSSI will be a good screening tool to assess the heat strain of workers exposed to sunlight or a hot environment at work before a precise and invasive assessment is conducted.

The forward and backward translations were conducted according to the measures recommended for cultural adaptation [22, 23, 41]. An expert committee which consists of various professionals related to occupational health as well as a representative of outdoor workers was consulted to review both versions of the translations as well as to evaluate the questionnaires quantitatively. Even though the team of developers of the original HSSI was not included in the expert committee, all of the identified discrepancies and inconsistencies were discussed, and amendments were conducted without affecting the meaning of the original items. The calculated I-CVI for all of the reviewed items and the S-CVI were 1.00 after item enhancements based on the experts’ recommendations and were reviewed for the second time by all the experts. Furthermore, the calculated I-FVI for all of the items and the S-FVI were equal to or more than the minimum value of 0.83 [26, 28]. These indicated that both the content and face validity of the Malay version of HSSI is acceptable.

Factor analyses using principal component analysis with Varimax rotation were conducted to extract the main factors of the 18-item HSSI. This procedure was chosen to evaluate the construct validity of the translated questionnaire by exploring the correlations among the items in the Malay version of HSSI to identify the variables’ underlying structure. No item was omitted during the translation process and psychometric testing.

There were only three main domains in the original HSSI, which were labelled as subjective judgment, protective clothes, and physical activities [42]. However, this study extracted four domains or factors from the PCA for the Malay version of HSSI. They were labelled as (i) Physical activity and perceived symptoms at work (seven items); (ii) Perception of workplace ambient (four items); (iii) Working attire and personal protective equipment (four items); and (iv) Personal observation of the workplace setting (three items). All of the factors extracted had an acceptable level of internal consistency reliability, with each factor having Cronbach’s alpha value of more than 0.70 [32]. The test-retest reliability of the HSSI scoring was also acceptable, with a good ICC of 0.792.

Item 6, 7, 8, 9, and 18 are loaded together with Item 5 and 17, which point to different understandings of these items in the Malaysian setting. Physical activity was combined with perceived symptoms at work. One probable explanation for this combination is the intensity of physical activity at work is connected and has effects on the symptoms experienced by the workers. Furthermore, items 10,11 and 12 were also loaded separately, and the new factor labelled ’Observation of the workplace setting’ was created for these items. One likely explanation was that most workers (86.2%) worked outdoors, and their interpretations were towards their outdoor working environments.

### Study limitations

There were a few limitations identified in the study. Even though the sample size of the field study fulfills the criteria of a minimum subject-to-item ratio of 10:1, as recommended by Hair et al. (2014) [30], it would be more appropriate to have a larger sample size to diminish error in data and ensure reliability for the factors extracted. Two-thirds of the workers were male workers who may not perceive heat as an occupational hazard as they were used to working long hours outdoors.

Structured interviews were conducted among participants who were illiterate or with a low level of education which was assisted by translators who were well versed in the local language (Bajau and Kadazan/Dusun). Therefore, social desirability bias could be considered as the workers may tend to give responses based on what was more appealing to them, which could be overrated or underrated, and the responses given were more favourable to the researcher rather than their actual experiences.

Most of the workers in the field study mainly worked outdoors, while participants who were supervising or guarding were working indoors and outdoors. No worker was specifically working in the hot indoor environment. Therefore, there may be a limitation in using this Malay version of questionnaire among those working in the hot indoor environment. Besides, the field study was mainly conducted among the outdoor workers in the Borneo state of Sabah and northern Peninsular Malaysia. Hence, further studies among Malay-speaking workers, especially those working in hot indoor environments, are crucial to validate the findings of this study.

## Conclusion

The findings from this study indicate that the Malay version of the Heat Strain Score Index is a valid and reliable instrument. Confirmatory factor analysis (CFA) will be need to be conducted once data is available from the actual study population to further add value to the construct validity, convergent validity, discriminant validity and composite reliability of the translated questionnaire. It can therefore be used to assess the heat strain among the Malay-speaking susceptible workers in Malaysia who are exposed to hot, humid environments. This ensures that further assessment and adequate control and prevention measures can be taken based on the level of heat strain identified.

## Supporting information

S1 File (PDF)

S2 File (PDF)
