# Supplementary material for: Translation and cross-cultural adaptation of heat strain score index (HSSI) into the Malay language
Source: PLoS One. 2023 Feb 22;18(2):e0281217. doi: 10.1371/journal.pone.0281217 (PMC9946246; doi:10.1371/journal.pone.0281217)
Supplement: S1 File — (PDF) [file pone.0281217.s001.pdf]

ResearchGate

See discussions, stats, and author profiles for this publication at: https://www.researchgate.net/publication/283675384

Development and validation of a questionnaire for preliminary assessment of heat stress at workplace

Article *in* Journal of Research in Health Sciences · January 2015

CITATIONS
17

READS
2,000

4 authors, including:

Habib Dehghan
Isfahan University of Medical Sciences
62 PUBLICATIONS **440** CITATIONS

SEE PROFILE

Mohammad Reza Maracy
Isfahan University of Medical Sciences
172 PUBLICATIONS **1,870** CITATIONS

SEE PROFILE

Some of the authors of this publication are also working on these related projects:

Project infectious diseases View project

Project A survey on the association of anxiety-depressive symptoms with vascular endothelial functions View project

All content following this page was uploaded by Habib Dehghan on 26 July 2016.

The user has requested enhancement of the downloaded file.

Appendix 1

Scale of Heat Strain Score Index (HSSI)

Instruction for use of Heat Strain Score Index:

1-Mark each question based on question of subject and your observation of the appropriate condition

2-When completed, for each question, write your score in the" primary score" column in Total Scores Calculation Sheet

3- Primary Score each question is multiplied by effect coefficient and final score recorded

4-Add the final scores of the Calculation Sheet for total score result

Questions

Q1 - How do you feel your workplace air temperature?

Very cold (-3)

Cold (-2)

Slightly Cool (- 1)

normal (0)

slightly warm (1)

Warm (2)

very warm (3)

Q2 - How do you feel the humidity level of your workplace?

Dry (a feeling of dryness in the mouth and throat) (-2)

Appropriate and desirable (0)

Wet skin (1)

Clothes sticking to the skin surface (2)

Fully wet skin (3)

Sweat loss from the skin surface (4)

Q3 - How do you feel the temperature of adjacent surfaces due to contact with your hands?

I feel too cold (-3)

I feel cold (-2)

I feel cool (-1)

I do not feel cold or hot (0)

I feel hot (1)

Their heat cannot be tolerable (2)

If my skin is in touch with them I will be burnt .(3)

Q4 - How do you feel the flow of air in your workplace?

The existence of cold weather circulation (-3)

The existence of cold weather current (-2)

Gentle stream of pleasing air (-1)

Sense of stability in the gentle flow of air or warm air (1)

The moderate flow of warm air (2)

Extreme current of hot weather (3)

Q5 - While you are working, the intensity of physical activity you do is like which of the following conditions?

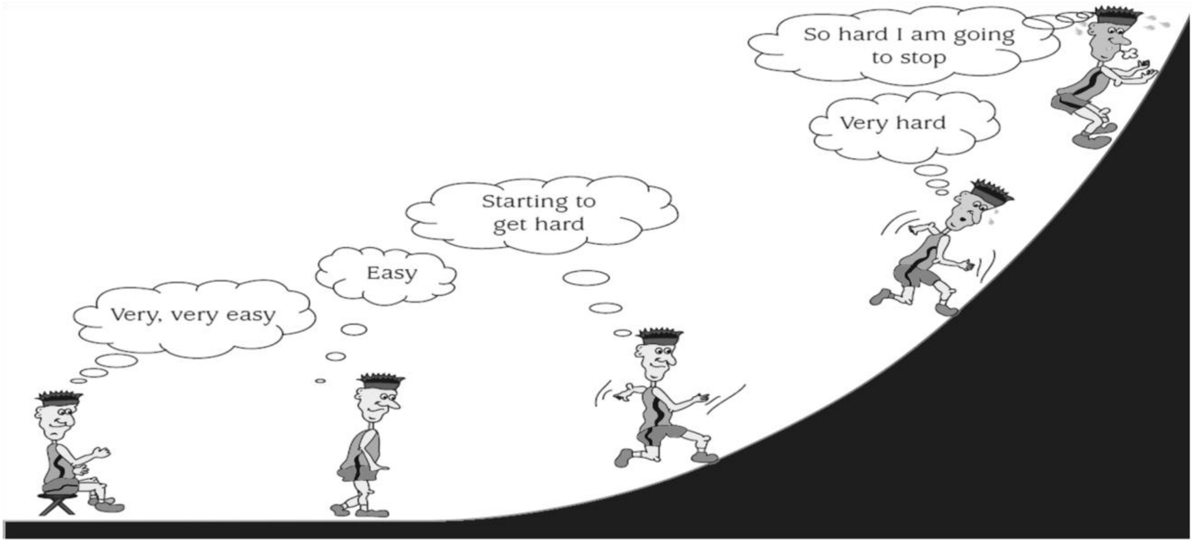

0	1	2	3	4

Q6 – How much is the amount of sweating throughout your working?

I do not feel like sweating (0)

I feel the sweat on the armpit and inguinal (1)

I feel the sweat on the chest and back (2)

Sweating is so severe that the underwear clothing get wet (3)

Sweating is so severe that I feel it on my face (4)

Sweating is so severe that it is flowing all over my body (5)

Q7 - How much fatigue you are at work?

I'm not tired at all (0)

I'm a little tired (1)

I'm tired (2)

I'm exhausted(3)

I'm so exhausted that I desire to have a break (4)

Q8 - How much is the intensity of your thirst when you are at work?

I don’t get thirsty (0)

I get a little thirsty (1)

I get thirsty (2)

I get very thirsty (3)

I get so thirsty that my mouth and throat get dry and they can’t be wet with saliva (4)

Q9 – How intensive you are suffering from heat?

I'm not annoyed (0)

I'm a little annoyed (1)

I'mannoyed (2)

I'm very annoyed (3)

I'm so annoyed that I want to quit my job posts (4)

Q10 - How do you feel about the size of working space within the building?

Spacious (0)

Appropriate common space (1)

Limited cramped space (2)

Q11 - How is the ventilation system in your workplace?

Active and high ventilation (-1)

Appropriate ventilation, it is not needed to be ventilated (0)

Inadequate ventilation (1)

Despite the lack of air conditioning, there is no ventilation (2)

Q12 - In which environments below you are doing your own tasks now?

Outdoors (0)

Indoor (2)

Both (1)

Q13 - What kind of clothes do you use while you work out?

T-shirts and jeans (no work clothing worm) (0)

Normal work clothing (underwear+ shirts and pants) (1)

Full suits (underwear+ work clothing coverall) (2)

Heavy or wool clothing or winter work clothing (underwear + double cloth coveralls) (3)

Water -proof clothing (chemical protective clothing, wind visor, leather) (5)

Fully enclosed suit with hood and gloves (7)

Q14 - What color is your work clothing?

Light colors (e.g. white, cream, yellow, light blue, orange, etc.) (0)

Dark colors (e.g. Black, dark brown, dark red and dark blue) (1)

Q15 - What material is your work clothing?

Cotton (1)

Cotton and synthetic fibers (2)

Fireproof and water proof (3)

Q16 –During the work, which equipment do you use including the following personal protection equipment?

Self-contained breathing apparatus (2)

Full- face respirator (1.5)

Half-face respirator (1)

Water proof boot (1)

Leather apron (1)

Anti-dust mask (0.5)

Face shield (0.5)

Not-cotton glove (0.5)

Helmet (0.5)

Ear muff (0.5)

Q17 – What is your more often body posture when you are at work?

Usually sitting (1)

Usually standing with low mobility (2)

Standing with a high mobility (3)

Usually I am Walking (4)

Q18 - Now which of the following symptoms do you have while you are working?

Mild headache (0.5)

Dizziness (0.5)

Weakness (0.5)

Muscle pain (0.5)

Red acne appearance (0.5)

Lower concentration (0.5)

None (0)

Calculation of Heat Strain score Index

Total Scores Calculation Sheet

**Number of Questions**	**Primary score**	**Effect coefficient**	**Final Score**
Q1		0.73	
Q2		0.67	
Q3		0.65	
Q4		0.61	
Q5		0.63	
Q6		0.67	
Q7		0.57	
Q8		0.84	
Q9		0.81	
Q10		0.28	
Q11		0.68	
Q12		0.31	
Q13		0.36	
Q14		0.29	
Q15		0.33	
Q16		0.50	
Q17		0.37	
Q18		0.57	
Total Score			

Evaluation result:

1- The total score which is less than 13.5 indicates that the person has no or low heat strain (Green Zone or safe level).

2- The total score between 13.6 to 18 indicated that there is a potential of heat-induced illnesses occurring and it is needed to further evaluation of heat stress more precisely (Yellow Zone or alarm level)

3- The total score greater than 18 indicated that the onsets of heat-induced illnesses are very likely and appropriate control measures should be taken as soon as possible to reduce heat strain (Red Zone or danger level).

View publication stats
