# Supplementary material for: Translation and cross-cultural adaptation of heat strain score index (HSSI) into the Malay language
Source: PLoS One. 2023 Feb 22;18(2):e0281217. doi: 10.1371/journal.pone.0281217 (PMC9946246; doi:10.1371/journal.pone.0281217)
Supplement: S2 File — (PDF) [file pone.0281217.s002.pdf]

## ***Translated Malay Version of Heat Strain Score Index (HSSI) with scale***

# INDEKS SKOR TEGASAN HABA

ARAHAN: Sila jawab semua soalan dengan menandakan pilihan jawapan yang paling sesuai dengan pekerjaan anda. **Jika anda merasakan tiada jawapan yang 'paling tepat', sila tandakan jawapan yang paling hampir sekali**.

Untuk soalan di bawah, sila tandakan (√) di dalam petak jawapan yang paling tepat/paling hampir sekali.

1. Bagaimana anda merasakan suhu di tempat kerja anda?

| | | | |
|---|---|---|---|
| Sangat sejuk (-3) | | Sedikit panas (1) | |
| Sejuk (-2) | | Panas (2) | |
| Sedikit sejuk (-1) | | Sangat panas (3) | |
| Normal /Biasa (0) | | | |

2. Bagaimana anda merasakan tahap kelembapan di tempat kerja anda?

| | | | |
|---|---|---|---|
| Kering [rasa kering di mulut dan tekak] (-2) | | Pakaian melekat pada permukaan kulit (2) | |
| Sesuai dan wajar (0) | | Kulit basah sepenuhnya (3) | |
| Kulit rasa lembap/basah (1) | | Kehilangan peluh dari permukaan kulit (4) | |

3. Bagaimana anda merasakan suhu pada permukaan berhampiran apabila bersentuhan dengan tangan anda?

| | | | |
|---|---|---|---|
| Saya berasa terlalu sejuk (-3) | | Saya berasa panas (1) | |
| Saya berasa sejuk (-2) | | Saya tidak tahan dengan kepanasan permukaan itu (2) | |
| Saya berasa sedikit sejuk (-1) | | Sekiranya kulit saya bersentuhan dengan permukaan itu, kulit saya akan melecur (3) | |
| Saya tidak berasa sejuk ataupun panas (0) | | | |

4. Bagaimana anda merasakan aliran udara di tempat kerja anda?

| | | | |
|---|---|---|---|
| Kewujudan peredaran udara sejuk (-3) | | Rasa aliran udara atau udara suam yang lembut dan stabil (1) | |
| Adanya arus udara sejuk (-2) | | Aliran udara suam yang sederhana (2) | |
| Aliran udara lembut yang menyenangkan (-1) | | Cuaca/ Arus udara panas yang melampau (3) | |

5. Semasa anda bekerja, apakah tahap intensiti aktiviti fizikal yang anda lakukan? (Contoh seperti dalam gambarajah di bawah)

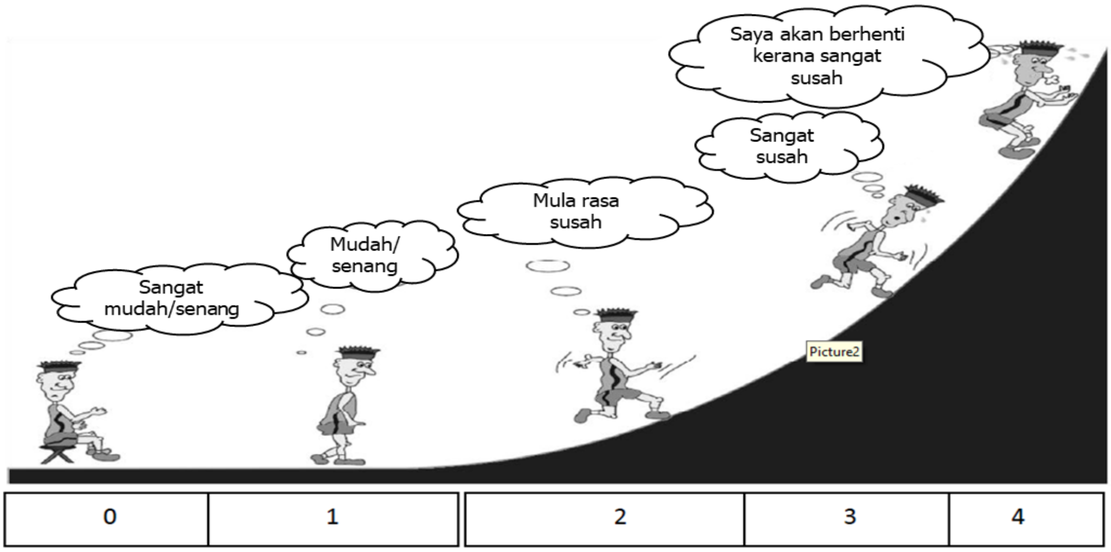


6. Berapakah tahap perpeluhan yang anda alami sepanjang anda bekerja?

| | | | |
|---|---|---|---|
| Saya tidak rasa saya berpeluh (0) | | Saya berpeluh sangat banyak sehingga pakaian dalam menjadi basah (3) | |
| Saya merasakan peluh di ketiak dan pangkal paha (1) | | Saya berpeluh sangat banyak sehingga saya dapat merasakannya di bahagian muka saya (4) | |
| Saya merasakan peluh di bahagian dada dan belakang (2) | | Saya berpeluh sangat banyak sehingga mengalir ke seluruh badan saya (5) | |

7. Berapakah tahap keletihan anda semasa anda bekerja?

| | | | |
|---|---|---|---|
| Saya tidak berasa letih/penat sama sekali (0) | | Saya berasa sangat letih/penat (3) | |
| Saya berasa sedikit letih/penat (1) | | Saya sangat letih/penat sehingga saya terpaksa berehat (4) | |
| Saya berasa letih/penat (2) | | | |

8. Berapakah tahap kehausan anda semasa anda bekerja?

| | | | |
|---|---|---|---|
| Saya tidak berasa haus /dahaga (0) | | Saya berasa sangat haus / dahaga (3) | |
| Saya berasa sedikit haus / dahaga (1) | | Saya berasa sangat haus / dahaga sehingga mulut serta tekak saya berasa sangat kering dan mereka tidak dapat dilembapi dengan air liur (4) | |
| Saya berasa haus / dahaga (2) | | | |

9. Apakah intensiti/tahap kepanasan yang anda tanggung / derita di tempat kerja?

| | | | |
|---|---|---|---|
| Saya tidak berasa terkesan (0) | | Saya berasa sangat terkesan (3) | |
| Saya berasa sedikit terkesan (1) | | Saya berasa sangat terkesan sehingga saya ingin berhenti kerja (4) | |
| Saya berasa terkesan (2) | | | |

10. Apakah pendapat anda tentang ukuran ruang tempat kerja di dalam bangunan?

| | |
|---|---|
| Luas (0) | |
| Ruang gunasama yang sesuai (1) | |
| Ruang sempit dan terhad (2) | |

11. Bagaimanakah sistem pengudaraan di tempat kerja anda?

| | | | |
|---|---|---|---|
| Sistem pengudaraan yang aktif dan tinggi (-1) | | Pengudaraan yang tidak mencukupi (1) | |
| Pengudaraan yang baik/sesuai, tidak perlu ada sistem pengudaraan (0) | | Walaupun kekurangan pendingin udara, tidak ada sistem pengudaraan (2) | |

12. Kawasan persekitaraan manakah anda melakukan tugas anda sekarang?

| | | | |
|---|---|---|---|
| Kawasan Luar (0) | | Kedua-duanya (1) | |
| Di dalam bangunan (2) | | | |

13. Apakah jenis pakaian yang anda pakai semasa bekerja?

| | | | |
|---|---|---|---|
| T-shirt dan seluar jeans (0) | | Pakaian tebal atau berbulu atau pakaian kerja untuk musim sejuk (seluar dalam + pakaian dua lapis) (3) | |
| Pakaian kerja biasa (pakaian dalam + kemeja dan seluar) (1) | | Pakaian kalis air (Pakaian pelindung kimia, penutup angin, pakaian kulit) (5) | |
| Pakaian kerja lengkap (pakaian dalam + pakaian kerja yang khas) (2) | | Sut tertutup penuh dengan tudung / sarung kepala dan sarung tangan (7) | |

14. Apakah warna pakaian kerja anda?

| | | | |
|---|---|---|---|
| Warna terang<br>(contoh: putih, krim, kuning,oren, biru muda dll) (0) | | Warna gelap<br>(Contoh: hitam, coklat tua, merah tua, biru tua dll) (1) | |

15. Apakah jenis bahan (*material*) pakaian kerja anda?

| | | | |
|---|---|---|---|
| Kapas (1) | | Tahan api dan kalis air (3) | |
| Kapas dan gantian sintetik (2) | | | |

16. Semasa anda bekerja, apakah peralatan yang anda gunakan termasuk peralatan perlindungan diri yang tersenarai di bawah?

| | | | |
|---|---|---|---|
| Alat pernafasan serba lengkap (2) | | Topeng anti debu /penutup muka fabrik (0.5) | |
| Alat pernafasan muka penuh (1.5) | | Pelindung muka (0.5) | |
| Alat pernafasan separuh muka (1) | | Sarung tangan jenis bukan kapas (0.5) | |
| But kalis air (1) | | Topi keselamatan (0.5) | |
| Apron kulit / apron plastik (1) | | Alat pelindung pendengaran (0.5) | |

17. Apakah postur/ posisi badan anda yang lebih kerap/ biasa semasa bekerja?

| | | | |
|---|---|---|---|
| Kebiasaannya saya duduk (1) | | Saya bekerja berdiri dengan mobiliti/ pergerakan yang tinggi (3) | |
| Kebiasaannya saya berdiri dengan mobiliti/pergerakan yang sedikit (2) | | Kebiasaannya saya berjalan semasa bekerja (4) | |

18. Apakah tanda / simptom seperti yang tersenarai di bawah yang anda alami semasa anda bekerja?

| | | | |
|---|---|---|---|
| Sakit kepala yang ringan (0.5) | | Penampilan jerawat merah / ruam panas (0.5) | |
| Pening (0.5) | | | |
| Letih lesu / lemah (0.5) | | Kurang tumpuan (0.5) | |
| Sakit otot (0.5) | | Tiada (0) | |
